# Characterization of carbapenem-resistant *Pseudomonas aeruginosa* clinical isolates, carrying multiple genes coding for this antibiotic resistance

**DOI:** 10.1186/s12941-014-0043-3

**Published:** 2014-09-02

**Authors:** Camila Rizek, Liang Fu, Leticia Cavalcanti dos Santos, Gleice Leite, Jessica Ramos, Flavia Rossi, Thais Guimaraes, Anna S Levin, Silvia Figueiredo Costa

**Affiliations:** Laboratory of Bacteriology of Department of Infectious, Diseases of University of São Paulo, Dr. Eneas Carvalho de Aguiar 470, São Paulo, Zip Code 02461011 Brazil; Department of Infectious, Diseases of University of São Paulo, Dr. Eneas Carvalho de Aguiar 470, São Paulo, Zip Code 02461011 Brazil; Laboratory of Microbiology of Hospital das Clinicas of University of São Paulo, Dr. Eneas Carvalho de Aguiar 255, São Paulo, Zip Code 02461011 Brazil

**Keywords:** Pseudomonas, Carbapenemases, KPC, VIM, SPM

## Abstract

**Background:**

Carbapenemase genes are one of the most frequent mechanisms reported in carbapenem-resistant *P. aeruginosa;* however, description of *P. aeruginosa* co-harbouring two or more carbapenemases is unusual.

**Methods:**

In this study we evaluated the presence of carbapenemase genes and the clonality of *P. aeruginosa* isolates obtained from a hospital over a 12-year period. A total of 127 isolates of carbapenem-resistant *P. aeruginosa* recovered from 109 patients feces (four samples), rectal swab (three samples), nasal swab (one sample) and anal abscess (one sample), were evaluated. Minimum inhibitory concentrations of the following antibiotics imipenem, meropenem and polymyxin E were determined by broth microdilution. The molecular profile of isolates was evaluated by pulsed field gel electrophoresis (PFGE). PCR for the following carbapenemase genes *bla*_IMP;_*bla*_SPM;_*bla*_VIM;_*bla*_SIM;_*bla*_NDM;_*bla*_KPC;_*bla*_GES_ and nucleotide sequencing to confirm the enzyme gene types were performed and compared with the database available on the Internet (BLAST-http://www.ncbi.nlm.nhi.gov/blast/).

**Results:**

All isolates were carbapenem-resistant, their MIC_50_ and MIC_90_ were respectively 64 μg/mL and 256 μg/mL to imipenem and 32 μg/mL and 256 μg/mL to meropenem, all isolates except one (MIC = 8 mg/L) were susceptible to polymyxin E. The most frequent carbapenemase genes identified were *bla*_SPM_ identified in 41 isolates (32%), followed by 10 with *bla*_kpc_ and 5 with *bla*_VIM_ (3.9%). All belonged to the class SPM-1 and VIM-2. In 2011, one isolate harbouring three carbapenemase genes (SPM-1, VIM-2 and KPC-2) that belonged to a new clone was identified in a hematopoietic stem cell transplanted patient. Then, 19 carbapenem-resistant *P. aeruginosa* were identified in an outbreak that occurred in the bone marrow transplant unit, all positive for SPM-1 gene, and 9 (47.3%) harbored both SPM-1 and KPC.

**Conclusion:**

Our findings showed that PCR for KPC gene should be performed to evaluate carbapenem resistance in *P. aeruginosa* and that this agent can harbor more than one carbapenemase gene. Attention should be focused on the possible rapid spread of KPC in *P. aeruginosa* isolates and for the fact that *P. aeruginosa* may become a reservoir of this transmissible resistance mechanism.

## Background

Carbapenem–resistant *P. aeruginosa* has become an important problem all over the world challenging the current diagnostic approaches. Carbapenemase genes are one of the most frequent mechanisms reported in carbapenem-resistant *P. aeruginosa* [1-5]. It is important to identify carbapenemase genes transmitted on mobile genetic elements which can lead to the spread of resistance of *P. aeruginosa* to carbapenem, which are the main drugs used to treat infections caused by this agent. In Brazil, the most common carbapenemase is the metallo-betalactamase, SPM, however, recently *P. aeruginosa* harboring KPC was identified [2].

In this study, we evaluated the presence of carbapenemase genes and the clonality of carbapenem- resistant *P. aeruginosa* isolates obtained from a teaching hospital over a 12-year period.

## Methods

### Study setting

The study was conducted in The *Central Institute of Hospital das Clinicas* (ICHC – FMUSP), Brazil, a teaching hospital with 1,000 beds, ten intensive care units totalizing 110 beds and a bone marrow transplant ward with 20 beds.

### Isolates

A total of 129 *P. aeruginosa* carbapenem-resistant clinical isolates identified over a 12-year period, from 1998 to 2012, recovered from 109 patients, hospitalized in the Clinical and Surgical nursery, Intensive Care, Burned, Haematology and Bone Marrow units at Hospital das Clinicas-FMUSP were evaluated.

### Susceptible profile

Minimum inhibitory concentrations (MICs) of imipenem, meropenem and polymixin E were determined by broth microdilution according with Clinical Laboratory Standards Institute (CLSI 2012).

### Molecular typing

Bacterial isolates were grown on blood agar overnight at 37°C. Gel blocks were made by using equal volumes of 2% low-melting-point agar (BioRad, USA) and a bacterial suspension of 9 × 10^8^ cells. Genomic DNA was digested with 10U of XBAI (Fermentas, USA), [6] (Sekiguchi ref). DNAs were separated by pulsed-field gel electrophoresis (PFGE) using a CHEF-DR III system (Bio-Rad, USA). Running conditions were 21 h at 14°C, with and initial switching time of 1 s and final time of 30 s, at 6 V/cm. PFGE patterns were interpreted according to Tenover *et al.* 1997 [7].

### Carbapenemases genes

PCR for the following carbapenemase genes *bla*_IMP;_*bla*_SPM;_*bla*_VIM;_*bla*_SIM;_*bla*_NDM;_*bla*_KPC;_*bla*_GES_ was done as described previously [8-11]. The following reference strains were used as control in this study*: P. aeruginosa* that produced IMP-1, VIM-2, SIM-1, SPM-1 [10], KPC ATCC and *E. coli* NDM. Nucleotide sequencing to confirm the enzyme gene types was performed by MegaBACE 1000. The sequences were analyzed using the software Sequence Analyzer with the Base Caller Cimarron 3.12. The genetic sequence was compared with the database available on the Internet (BLAST-http://www.ncbi.nlm.nhi.gov/blast/). The KPC sequence was also comparing with KPC lahey databases (http://www.lahey.org/Studies/other.asp#table1).

## Results

**Figure 1:**
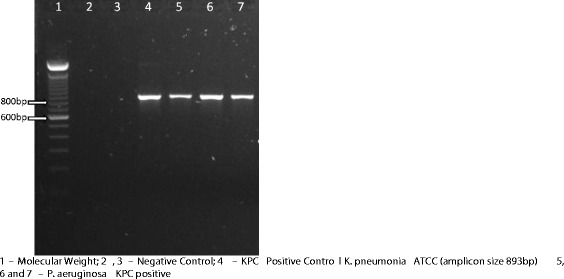
**PCR for detection of SPM gene in P. aeruginosa carbapenem-resistant isolates.** 1 - SPM Positive Control (amplicon size 798 bp). 2, 3 and 4 - P. aeruginosa SPM positive; 5, 6 - Negative Control and 6 - Molecular Weight.

**Figure 2:**
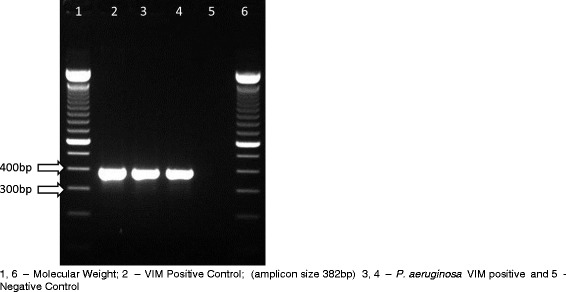
**PCR for detection KPC gene in** ***P. aeruginosa*** **carbapenem-resistant isolates.** 1 – Molecular Weight; 2, 3 – Negative Control; 4 – KPC Positive Control (amplicon size 893 bp); 5, 6 and 7 – *P. aeruginosa* KPC positive.

**Figure 3:**
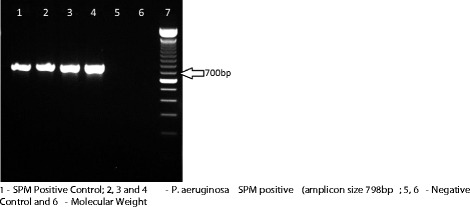
**PCR for detection of VIM gene in** ***P. aeruginosa*** **carbapenem-resistant isolates.** 1, 6 – Molecular Weight; 2 – VIM Positive Control (amplicon size 382 bp); 3, 4 – *P. aeruginosa* VIM positive and 5 - Negative Control.

All isolates were carbapenem-resistant, their MIC_50_ and MIC_90_ were respectively 64 μg/mL and 256 μg/mL to imipenem and 32 μg/mL and 256 μg/mL to meropenem. All isolates except one (MIC: 8 μg/mL), were susceptible to colistin, MICs varied from 0.25 to 2 μg/mL. They were recovered from blood (120 samples), feces (four samples), rectal swab (three samples), nasal swab (one sample) and anal abscess (one sample). PFGE showed that, from 1998 to 2009, 25% (27 of 108 strains) belonged to one predominant clone (A1), thirty-two (A2) of 108 isolates (29.7%) were closely related to them and sixteen (14.8%) were possibly related (A3, A4, A5, A6 and A7) to the predominant clone (A1). Thirty-three (30.5%) showed no relation with the predominant clone A1. Among the 129 isolates 50 (39%) harboured a carbapenemase, the most frequent carbapenemase genes identified were *bla*_SPM_ identified in 41 isolates (32%) (Figure 1), followed by 10 with *bla*_kpc_ (Figure 2) and 5 with *bla*_VIM_ (Figure 3). GES-5 was identified only in 3 isolates from one burned patient. *Pseudomonas aeruginosa* harbouring SPM-1 was identified for the first time in the Bone Marrow Transplant unit in 1998, VIM-2 in the Emergency Room in 2001 and GES-5 in the Burned Intensive Care Unit. GenBank accession numbers: JX840596 (VIM-2), JX682700-JX682705 (KPC-2) and JX870518-JX870528 (SPM-1).

In 2012 during an outbreak that occurred in the bone marrow transplant unit, 19 carbapemem-resistant *P. aeruginosa* were identified (clone K, L, L1, L2, L3 and M), all positive for SPM-1 gene, and 10 (47.3%) harboured both SPM-1 and KPC-2. The majority of the KPC, six isolates of nine, belong to the clone of the outbreak (K).

The relation between molecular profile and carbapenemase gene for all period is shown on Table 1.

**Table 1:** **Carbapenemase genes and clonality of 129 carbapenem-resistant** ***P. aeruginosa*** **isolated in Hospital das Clinicas, over a 12-year period**

**Carbapenemase gene**	**Number of isolates**	**PFGE clone**	**Year of identification**
None (n = 79)	27	A1	1998-2008
	25	A2	
	2	A3	
	5	A5	
	8	B	
	6	C	
	2	D	
	2	E	
	2	F	
SPM-1 (n = 33)	7	A2	
	3	A3	
	1	A4	
	1	A6	
	1	A7	
	6	G	1998-2012
	2	I	
	6	L	
	2	L1	
	1	L2	
	1	L3	
	2	M	
VIM-2 (n = 4)	1	B	
	1	D	
	2	H	2001-2009
GES-5 (n = 3)	3	A5	
			2001
SPM-1 and KPC-2 (n = 9)			
	6	K^B^	
	1	L^C^	
	1	L1^D^	2012
	1	M^E^	
SPM-1 and VIM-2 and KPC-2 (n = 1)	1	J^A^	2011

## Discussion

It is the first report of *P. aeruginosa* co-harbouring *bla*_KPC_ and *bla*_SPM_ genes and the first report in Brazil of *P. aeruginosa* carrying KPC-2, VIM-2 and SPM-1. SPM-1 was the most frequent carbapenemase identified in our hospital, followed by KPC-2. KPC in *P. aeruginosa* is rare and occurs mainly in the American continent [1-5,12]. Recently, *P. aeruginosa* harbouring KPC was described in Argentina [13] and Iran [14], showing the potential rapid dissemination of this mechanism of resistance to the world.

Even though *P. aeruginosa* harbouring KPC was identified in 2010 in Brazil [2], no other report was published since then.

Only 50 of 129 *P. aeruginosa* carbapenem-resistant harboured a carbapenemase evaluated in this study. Thus, the carbapenem resistance could be related to other mechanism of resistance such as outer-membrane protein alteration, efflux system overexpression or new carbapenemase not yet identified [15-17].

*Pseudomonas aeruginosa* co-harbouring carbapenemase is uncommon; there are few reports in the literature [1;3]. *Pseudomonas aeruginosa* isolate co-harbouring KPC and a metallo-β-lactamase (IMP-8) was recently reported in Puerto Rico [3] and isolates co-harbouring KPC and VIM gene were identified in Colombia [1]. We described a new clone of *P. aeruginosa* co-harbouring SPM-1 and KPC-2 that caused an outbreak in a Bone Marrow transplantation unit, and an isolate co-harbouring three carbapenemase (SPM-1; KPC-2 and VIM-2) that belonged to a different clone then previous described in two outbreaks that occurred in this unit and were controlled with reinforcement of hand hygiene and contact precautions, one due to *P. aeruginosa* harbouring SPM-1 and other harbouring VIM-2 [18].

This *P. aeruginosa* isolate harbouring KPC-2 was identified for the first time in our hospital in 2011 in a hematopoietic stem cell transplanted patient. It harboured three carbapanemase genes (SPM-1, VIM-2 and KPC-2), and belonged to a new clone (J^A^) not identified before in the hospital. This isolate showed a resistant profile to both imipenem and meropenem with MIC of 64 μg/mL and 32 μg /mL, respectively, but was susceptible to polymyxin and colistin with a MIC of 2 μg /mL for both drugs.

In 2012 during an outbreak that occurred in the Bone Marrow Transplant unit, 19 carbapemem-resistant *P. aeruginosa* were identified (clone K, L, L1, L2, L3 and M), all positive for SPM-1 gene, and 10 (47.3%) harbored both SPM-1 and KPC-2. The majority of the KPC, six isolates of ten, belong to the clone of the outbreak (K^B^). Our data showed different clones circulating in our hospital and a new one predominate clone harboring KPC. A recent study also described dissemination of a new clone of *P. aeruginosa* harbouring KPC in a hospital in Argentina after a *K. pneumonia-*KPC positive outbreak [13].

Other interesting findings of our study are that GES-5 was restricted to the Burned Intensive Care Unit and was identified only in 2001. VIM-1 was first identified in the Emergency Room and then in the Bone Marrow Transplant Unit, and SPM is spread in different units in the hospital. These results were similar with previous Brazilian reports that showed that SPM-1 is endemic in several hospitals in the country [19,20].

The complete sequence of two KPC-harbouring plasmids, Plasmid pCOL-1 (31 529 bp), IncP-6 replicon group and Plasmid pPA-2 (7995 bp) from *P. aeruginosa* showed that they differing in size and in incompatibility group, and harbouring different genetic structures containing the blaKPC-2 genes [21]. These findings suggest that the carbapenemase resistance dissemination due to KPC in *P. aeruginosa* will be similar to that seen in Enterobacteriaceae. Thus, it is very important to understand the epidemiology of these multiresistant isolates, in order to achieve early implementation of adequate control measures to contain and reduce their dissemination in the hospital setting. *Pseudomonas aeruginosa* can acquired this transmissible resistance mechanism, going unnoticed and be a source of spread of KPC to other genus and species of bacteria. Besides carbapenem-resistance in *P. aeruginosa* can be due to two or more carbapenemase genes, including KPC-gene.

In conclusion, our findings showed that SPM-1 is the most frequent carbapenemase identified in our hospital, followed by KPC-2. Thus, PCR for KPC gene should be performed to evaluate carbapenem resistance in *P. aeruginosa* and this agent can harbor more than one carbapenemase gene. Attention should be focused on the possible rapid spread of KPC in *P. aeruginosa* isolates and for the fact that *P. aeruginosa* may become a reservoir of this transmissible resistance mechanism.
